# Am I losing it?

**DOI:** 10.3402/jchimp.v2i3.19167

**Published:** 2012-10-15

**Authors:** Norman M. Mann

**Affiliations:** Department of Medicine, University of Connecticut School of Medicine, Farmington, CT, USA

**Keywords:** cognitive, neurogenesis, structural imaging

## Abstract

Complaints of memory loss are frequent as one ages. Individuals worry about the presence of Alzheimer’s disease, but the presence of other intact intellectual abilities is reassuring to these people. We do not know the cause of Alzheimer’s disease. There are now 5.4 million confirmed cases in the United States. We know that the disease is an age-related, non-reversible brain disorder that develops over many years. Investigation is helped with the use of structural imaging (magnetic resonance imaging or computed tomography). Functional imaging is also valuable. Therapy for the problem involves various drugs; these help but do not cure the difficulty.

This is a question that most people recognize as dealing with the ability of the brain to function properly. Individuals get anxious when their memory becomes faulty. Often as one ages, complaints of ‘I can’t remember my name’ or ‘I don’t remember where I put my keys’ are heard, and this sort of experience causes insecurity.

Now, it is true that because of declines in death rates from hypertension, heart disease, diabetes, and cancer that our life expectancy in the United States has increased on the average to 78 years. Actually, women can expect in this country to live to age 81, five more years than men. In Japan, life expectancy has been estimated at 82 years.

With the longer life span, complaints of memory deficits have become more frequent. It is recognized that one’s capacity to memorize, retain new information, and remember names grows less and less as one ages, especially in those individuals over 70. It is an old story, sometimes humorous but at other times embarrassing, that you met someone who greets you warmly and makes some personal remarks, and you are at a loss because you do not remember their names, or of course, the person who comes up to you and says, ‘You remember me, certainly. What’s my name?’ and you stammer and mumble some inappropriate response. Or, you often try to handle the problem by saying, ‘Of course I remember you. How is everything?’ and then you try feebly somehow to introduce the acquaintance to your companion if there is one with you.

It is situations like these that cause panic and anxiety, and yet, while memory ability in these instances is obviously faulty, the fact that other intellectual abilities are satisfactory and one is able to perform well at work and at home should reassure individuals that Alzheimer’s disease has not yet made its appearance. Some investigators have termed this type of disturbance as ‘age-associated memory impairment’.

This type of complaint is one of the most common problems that are presented to a doctor, yet so many patients are reluctant to bring it up. A husband will often say to a doctor, ‘Everything is fine’ when suddenly, his wife speaks up and states, ‘No, everything is not fine. He is frequently forgetful and cannot remember names. Is he getting Alzheimer’s?’ Please test him, doctor’.

Memory is an intricate function that has three outstanding components: (a) Registration that has to do with attention and perception; (b) retention; and (c) remembering. There are different parts of the brain that control the abilities listed above. There are some simple tests that a skilled examiner could use to answer a request to ascertain a patient’s status.

To test recent memory, three objects can be named, and an individual is requested to repeat them to make sure that he or she is paying attention. They are told there will be retesting again in 5 or 10 min. In the meantime, other things are discussed so that the mind of the person is on other matters. Retesting occurs, and there should be a 100% accuracy result.

In addition, a patient should be able to repeat 100 backwards for at least ten numbers. Questions as to yesterday’s news events, the name of the president, and who preceded him can be quite illuminating.

Long-term memory is tested by asking such questions as date of birth, the name of the schools attended, and the state where the individual was born. A more detailed mini-mental status examination used by physicians can also be used. Normally aging people react at a much higher level than patients with Alzheimer’s disease.

We do not know what causes Alzheimer’s disease. Unfortunately, some cases occur at an early age and are usually due to an inherited genetic mutation. However, most cases occur after the age of 60. There are now 5.4 million confirmed cases in the United States, and by the year 2050, there may be 13 million. The difficulties associated with this disease make it the nation’s third most expensive medical problem. It has been estimated that the risk at age 80 is 1.5% within the next year and 3% at age 85 (1).

Regarding diagnosis, certainly a thorough history, examination, and laboratory tests are essential. A mental status evaluation has already been mentioned. Neuropsychologic studies are very helpful. Inquiry and investigation as to the presence of any smell sensation defect is important since one of the earliest manifestations of Alzheimer’s is smell impairment (2, 3). Taste sensation diminishes with age, although the degree is not as significant as in olfaction (4).

Alzheimer’s disease is an age-related non-reversible brain disorder that develops over a period of years. Initially, people develop memory loss and confusion, which could be mistaken for the memory changes that frequently occur with normal aging. However, behavior and personality defects develop associated with a decline in cognitive abilities and, eventually, significant loss of mental function follows. These problems are due to the deterioration of the connections between certain neurons in the brain and their destruction (5).

Early detection of Alzheimer’s disease is enhanced with the use of structural imaging with magnetic resonance imaging (MRI) or computed tomography (CT). This approach is essential to rule out other conditions similar to Alzheimer’s, but, of course, they require different therapy. Structural imaging can reveal tumors, the presence of strokes, problems from head trauma, or the development of fluid in the brain (6).

Functional imaging reveals whether the metabolism of cells in different portions of the brain is satisfactory. This process involves the use of position emission tomography (PET) and functional MRI (fMRI). Molecular imaging employs radiotracers to pick out cellular changes involved in certain diseases. This approach includes the use of PET, fMRI, and single photon emission computed tomography (SPECT).

Early studies point out that initially in Alzheimer’s disease, there may be changes in cerebrospinal fluid proteins. TAU and beta-amyloid are two proteins that contribute to abnormal brain deposits that are thought to be related to the disease.

Investigation has also possibly implicated several genes that involve risk for the development of Alzheimer’s disease. Therefore, going forward, genetic profiling may be a necessary part of the workup involved in assessing whether Alzheimer’s disease is present (7).

The primary question and one most frequently asked by individuals has to do with preventive measures. What can one do to avoid dementia? Experimentation on rodents in the laboratory has shown that neurogenesis (new nerve pathways) is stimulated when the environment improves. When the mother lavishes care on her offspring, the brain responds. When rodents are exercised daily, new nerve elements in the brain are produced as compared to rodents who are kept in a sedentary type of situation (8).

Similarly, in humans, exercise and brain stimulation (reading, use of the internet, crossword puzzles, art work, and woodworking) can prevent the much feared onset of brain deterioration and the inability to carry on the necessary duties involved in the activities of daily living.

The question of medication often arises in cases of cognitive impairment and actual dementia. There can be difficulty in arriving at the proper diagnosis. As Ronald Peterson of the Mayo Clinic states in *The New England Journal of Medicine* (June 9, 2011), ‘The key distinction between mild cognitive impairment and dementia pertains to the degree of functional impairment’. Dr. Peterson adds that ‘at present, there is insufficient evidence to recommend treatment with vitamin B supplementation in mild cognitive impairment. The role of homocysteine and folate is complex, and its clinical significance varies across the literature’.

Other treatments for Alzheimer’s disease have been suggested and are available. These, however, do not cure the problem. They merely retard the symptom process but in doing so, character of life is changed for the better. There has been emphasis on the use of anticholinesterase inhibitors for involved patients. Mark D’Esposito, MD, and Adam Gazzaley, MD, have spoken of the moderate effects of these drugs ‘perhaps because Alzheimer’s affects multiple neurotransmitters’. They state further that no medication exists for an age-associated memory decline (9). Furthermore, there are side effects to these drugs that can cause patients distress. Fatigue, insomnia, syncope, and indigestion are occasional difficulties that patients encounter.

FDA approved drugs for use in Alzheimer’s problems include donepezil (Aricept), galantamine (Razadyne), memantine (Namenda), rivastigmine (Exelon), and tacrine (Cognex) (10). To understand how these drugs work, one has to visualize a large communication network in which neurons are very active. Neurotransmitters transfer ‘messages’ from one cell to another. Alzheimer’s disturbs the mechanism and eventually destroys neurons. Donepezil, galantamine, rivastigmine, and tacrine are cholinesterase inhibitors, and these slow up the process that impairs the neurotransmitters. Memantine, however, governs the activity of glutamate, which is important for learning and memory. When cells are injured in Alzheimer’s disease, glutamate is produced in significant amounts, and cell destruction can occur.

It should be mentioned that tacrine can cause liver complications, so it is not commonly used.

Cost should be mentioned. The average cost per patient for one of these drugs is about $5 per day, or about $1800 per year. Results vary. The most significant change may occur in the first few months of therapy, although some benefits remain for several years.

It is of great interest that Wang and his associates at the Department of Neurology at Yale University have demonstrated in working with animals regarding memory loss that ‘restitution of the proper neurochemical environment can partially restore physiological integrity. The data establish that cognitive changes with age are malleable, and there is potential to restore at least some cognitive abilities in the elderly’ (11).

Wang showed that guanfacine (Intuniv, Tenex), a drug used for attention deficit hyperactivity, can enhance working memory in aged animals when administered systemically. On the basis of these data, Wang states that ‘guanfacine is currently being tested in elderly humans with PFC (prefrontal cortex) cognitive deficits’.

## Case report

A patient, 77 years of age, was seen because of significant decline in cognitive and memory function. A thorough examination had been done elsewhere, and neurologic studies including MRI of the brain were satisfactory. Memory testing showed significant impairment.

The patient was urged to see his neurologist again, and it was recommended that the patient be placed on Ritalin. This was done. The patient is now on 10 mg twice daily, and there have been remarkable changes for the better. The patient had exhibited slow motion in his activities, sleepiness, and episodes of dozing off. The patient is now more alert, talkative, and sharp in his responses. His memory appears also to have improved 25% since testing has confirmed this. The use of Ritalin in cases of the type described should be considered, but clearance from a cardiologist is recommended.

In summary, dementia is a syndrome of acquired cognitive defects sufficient to interfere with social or occupational functioning that results from various central neurodegenerative and ischemic processes. One of the most common types of dementia is Alzheimer’s disease. Again, there is no cure for dementia. Drug intervention, as mentioned before, improves symptoms, but the progression of the pathology is not affected.

Editor’s Note: As an internist in his 90s, still practicing and writing papers, it is reassuring to know that the author is not necessarily losing it.
